# Chronic stroke survivors underestimate their upper limb motor ability in a simple 2D motor task

**DOI:** 10.1186/s12984-024-01471-1

**Published:** 2024-10-01

**Authors:** Sebastian Sporn, M. Coll, S. Bestmann, N. S. Ward

**Affiliations:** Department of Clinical and Movement Neuroscience, Queens Square Institute of Neurology, UCL, London, UK

**Keywords:** Stroke, UL motor ability, Confidence, Non-use

## Abstract

**Background:**

Stroke survivors can exhibit a mismatch between the actual motor ability of their affected upper limb and how much they use it in daily life. The resulting non-use of the affected upper limb has a negative impact on participation in neurorehabilitation and functional independence. The factors leading to non-use of the affected upper limb are poorly understood. One possibility is that non-use comes about through inappropriately low confidence in their own upper limb motor abilities.

**Objective:**

We asked whether chronic stroke survivors underestimate the motor ability of their affected upper limb.

**Methods:**

20 chronic stroke survivors (Mean FM: 28.2 ± 10.5) completed a 2D reaching task using an exoskeleton robot. Target sizes were individually altered to ensure success rates were similar for both upper limbs. Prior to each reaching movement, participants rated their confidence about successfully hitting the target (estimated upper limb motor ability).

**Results:**

Confidence ratings were significantly lower for the affected upper limb (estimated ability), even though it was equally successful in the reaching task in comparison to the less affected upper limb (actual ability). Furthermore, confidence ratings did not correlate with level of impairment.

**Conclusions:**

Our results demonstrate that chronic stroke survivors can underestimate the actual motor abilities of their affected upper limb, independent of impairment level. Low confidence in affected upper limb motor abilities should be considered as a therapeutic target to increase the incorporation of the affected upper limb into activities of daily living.

## Introduction

Stroke is the leading cause of long-term neurological disability worldwide [1, 2]. A major contributor to individual disability is impairment of the upper limb, which is seen in upto 75% of stroke survivors [3–5]. Optimal recovery of the upper limb requires that it is regularly incorporated into activities of daily living and failure to do so will slow down recovery. Identifying factors that contribute to lower than expected levels of upper limb use will help identify much needed therapeutic targets.

Confidence about succeeding at a motor task is important for maintaining our daily interactions with the environment. Consequently, there is interest in whether some survivors incorporate the affected upper limb into daily life less than would be expected given their level of impairment, something often referred to as ‘non-use’ [6–9]. Upper limb ‘non-use’ in everyday life can restrict participation in meaningful daily activities and social interactions [10] and increase the risk of long-term deterioration in upper limb motor ability [11]. Characterising ‘non-use’ of the upper limb after stroke will lead to improvements in both recognition and treatment of this important clinical problem.

A potential mismatch between estimated (‘what I think I can do’) and actual (‘what I can actually do’) upper limb ability has been found in some [12–17], but not other [18–21] studies. These studies all characterised non-use as a difference between impairment measures (e.g. FMA-UL scale) and retrospective self-reported assessments of day to day upper limb use (e.g. Stoke Impact Scale) in stroke survivors with only mild impairment (FMA-UL > 50). A more thorough characterisation of post-stroke upper limb non-use might be possible by (i) assessing estimated and actual upper limb ability within the same objective assessment framework, and (ii) investigating stroke survivors with at least moderate or even severe impairment who also seem likely to suffer reduced confidence in affected upper limb motor ability.

Here, we revisit the issue of confidence about upper limb ability in chronic stroke survivors through the design of a novel 2D planar reaching task that can assess both actual and estimated upper limb ability in stroke survivors with a wide range of upper limb motor impairment. This experimental design enabled us to address the following questions in chronic stroke survivors: (i) Is confidence in achieving task success the same in the more and less affected upper limb? (ii) How is confidence about upper limb task success related to actual task performance? (iii) Is confidence about upper limb task success and actual task performance related to clinical measures including upper limb impairment?

## Methods

### Participant recruitment

20 chronic stroke survivors (≥ 6 months from stroke onset) were recruited from the Queen Square Upper Limb rehabilitation programme (QSUL) for the experiment. The inclusion criteria for this experiment were: (1) first ever stroke and (2) no other brain injury, neurological condition or major psychiatric illness, while exclusion criteria were: (1) hemi-spatial neglect or hemianopia, (2) severe aphasia, or (3) pain limiting ability to participate in tasks or follow the study protocol. All participants were comprehensively informed about the study, and written, informed consent was obtained before their participation. Consent was obtained for all forms of personally identifiable data including clinical and kinematic data. The study was performed in accordance with the Declaration of Helsinki and was approved by the UCL/UCLH Joint Research Office (UCL Project ID Number: 17/0209; IRAS Project ID Number: 222832). The study was supported by a grant from the Jon Moulton Trust Charity.

### Experimental apparatus for assessing upper limb kinematics

**Fig. 1:**
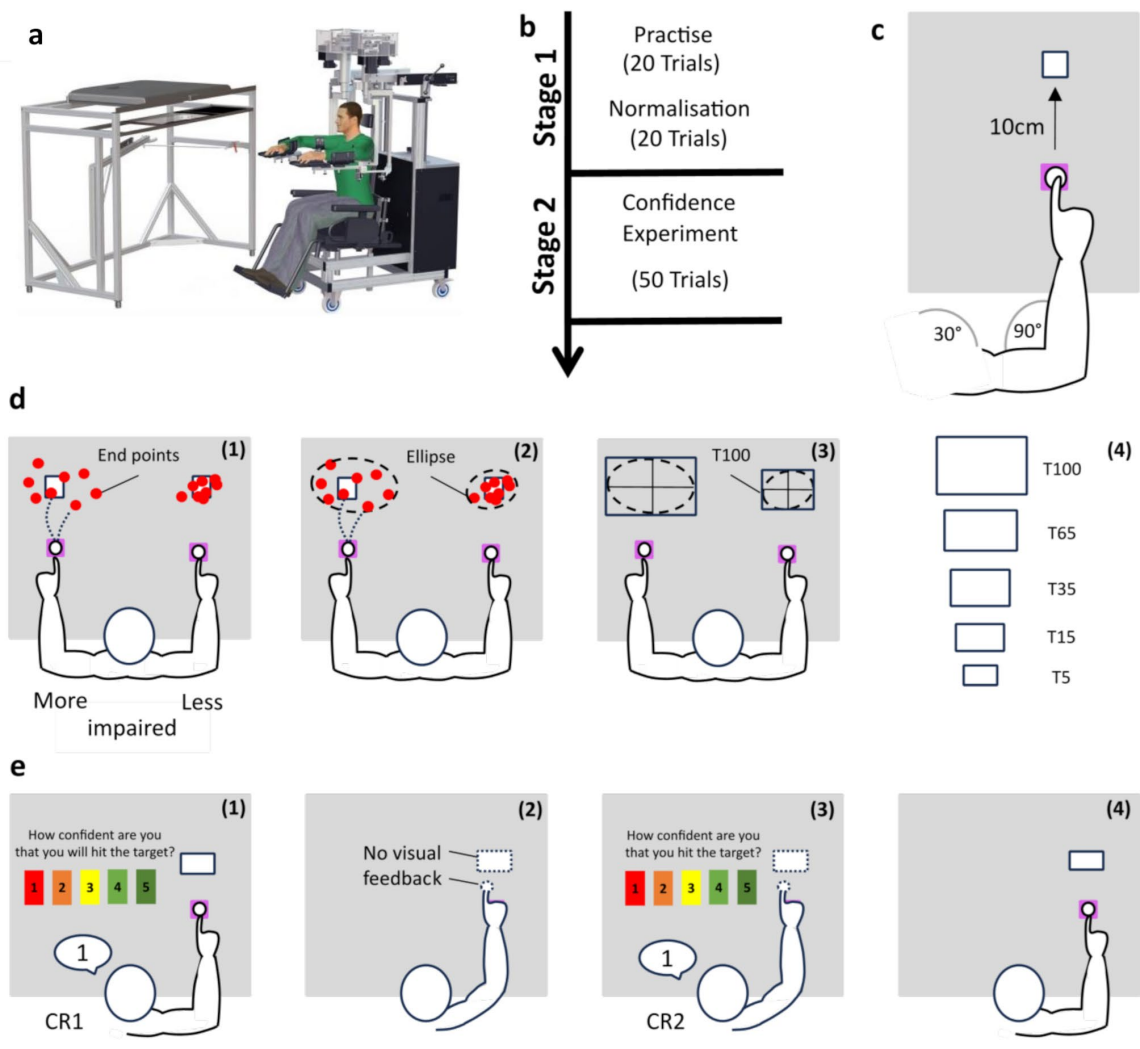
Experimental design **a**) Illustration of the KINARM Exoskeleton, which is a robot which gathers kinematic data from the upper limbs during task performance. **b**) Experimental stages. Stage 1 involves Practise and Normalisation (of upper limb motor ability – see 1d), while Stage 2 involves the Confidence experiment (see 1e). **c**) Illustration of the workspace and of the limb configuration. Participants were asked to make 10cm reaching movements from a start target to a peripheral target. Both were aligned so that the start target was at the midpoint of each limb’s workspace (90° elbow flexion and 30° shoulder flexion). **d**) Illustration of the Normalisation procedure. During the second part of Stage 2 participants made 20 reaching movements but without visual feedback of both the cursor and the peripheral target once they exited the start target. Reaching ability was normalise using the following procedure: (1) Determine reaching end points of each trial, (2) build a confidence ellipse with 99.9% confidence interval around the scatter, (3) Obtain the target that has ~100% success likelihood (T100; i.e., participants would have hit this target in almost every trial), (4) Create targets based on T100 with increasing difficulty level (T65 – T5). **e**) Illustration of a trial during the Confidence Experiment. Participants were presented with one of the 5 targets determined during Normalisation. (1) Prior to reaching participants were asked to rate how confident they were in hitting the target (Confidence Rating 1 – CR1), (2) After logging the verbal report participants aimed for the target without visual feedback of both the cursor and target, (3) After 2000ms participants were asked to rate how confident they were that they hit the target (Confidence Rating 2 – CR2), (4) After logging the verbal report, visual feedback was turned on again and participants could start a new trial by moving the cursor into the start target.

The experiment was run using the KINARM Exoskeleton (BKIN Technologies Ltd, Kingston, ON, Canada); a robot which gathers kinematic data from the upper limbs during task performance. The KINARM Exoskeleton is a robotic device that supports the user’s limbs, forearms and hands, allowing only for horizontal movements involving shoulder and elbow joint flexion and extension (Fig. 1a). Participants sit with their limbs extended outward in a horizontal plane, typically at an angle of 85–90 degrees to the shoulder. The device includes an exoskeleton for each limb segment, customized for comfort and support. It also features a 2-D virtual reality display in the same plane as the limbs, providing visual targets and feedback. A calibration process before each session ensures accurate tracking and interaction within the virtual environment. Importantly, the robot provides gravitational support but does not assist in task completion in the experiment [22–24].

### Experimental procedure and design

Prior to the start of the experiment consent was obtained from all participants. Participants underwent a one-time calibration process to accurately capture 2D movements in space. The experiment consisted of 2 stages: **Stage 1** involved *Practise* and *Normalisation* (of motor ability), while **Stage 2** included the *Confidence Experiment* (Fig. 1b) which were completed with both the more and less affected limb. The order was counterbalanced across participants. Each part was verbally explained thoroughly to the participants before the start of each experimental phase. In total, the experiment lasted for approximately 35–40 min which included breaks between experimental parts and when moving from one limb to the other.

#### STAGE 1

In both *Practice* and *Normalisation* participants were asked to make reaching movements ‘as fast and accurately as possible’ to move a cursor (0.4 cm [2]) from a start target (2 cm [2]) to a peripheral target (1 cm [2]). The start target was positioned at the midpoint of each limb’s workspace (90° elbow flexion and 30° shoulder flexion), while the peripheral target was displaced 10 cm vertically along a straight line from the start target (Fig. 1c). The peripheral target appeared 300ms after participants moved into the start target. The start target turned green after a further 500ms which served as a Go cue. Participants started a new trial by moving their cursor back into the start target.

*Practice –* Provision of visual feedback of both cursor and target (vision ON).

Practice was included to familiarise participants with the task. Here participants had unlimited time to reach the peripheral target, which would disappear 300ms after it being reached. More specifically, participants were instructed to move their cursor ‘as fast and accurately as possible’ to the peripheral target. Importantly, constant visual feedback of both the cursor and targets was provided during each trial. In total participants completed 20 trials (each limb).

*Normalisation* of motor ability – No visual feedback of both cursor and target (vision OFF).

*Normalisation* was identical to the *Practice* phase except that visual feedback of both the cursor and the target disappeared once participants exited the start target. Participants were instructed to move their cursor ‘as fast and as close as possible’ to the peripheral target ‘without correcting your position after stopping’ (i.e., a single ballistic movement). Trials ended 2000ms after presentation of the Go cue. Importantly, participants did not receive any performance-based feedback (i.e., no binary feedback about task success and no visual feedback about target distance by showing the peripheral target again). Participants were asked to complete 20 no vision trials, which were used to normalise task success across participants and across more and less affected limbs as follows (Fig. 1d):

1) Determine reaching variability: The reaching movement end point (i.e., once reaching speeds fell below 2.0 cm/s) of each trial was determined (Fig. 1d (1)). Subsequently, a confidence ellipse with a confidence interval of 99.9% was calculated for the end point scatter to capture total reaching variability (Fig. 1d (2)).

2) Calculate target with 100% success rate: The area of the confidence ellipse represents the size of the peripheral target that would have yielded a ~ 100% success rate.

3) The width and height of this confidence ellipse was determined to create a new target with a predicted 100% success rate (T100) for both the more and less affected limb (Fig. 1d (3)). This procedure normalises reaching success across limbs for each participant.

4) Four further targets with success likelihoods of 65% (T65), 35% (T35), 15% (T15), and 5% (T5) were created (Fig. 1d (4)). To this end, 65%, 35%, 15%, and 5% of the height and length of T100 were calculated to create targets that have a 65%, 35%, 15%, and 5% success likelihood based on the original reaching end point scatter.

Note here that ‘confidence ellipse’ refers to a technique to determine the size of a scatter of data points with a pre-specified level of confidence. The confidence ellipse with a confidence interval of ~ 100% was used here to determine endpoint variability during reaching and is not related to participants confidence about task success.

#### STAGE 2

*Confidence experiment –* No visual feedback of either cursor and target (vision OFF).

We then investigated whether confidence about task success (estimated upper limb motor ability) matches actual task success (actual upper limb motor ability). Participants were not explicitly informed that their actual motor ability was normalised. Instead, participants were again instructed to move their cursor ‘as fast and as close as possible’ to the peripheral target ‘without correcting your position once having stopped the movement’, but this time without visual feedback of both the cursor and the target. (T100, T65, T 35, T15 or T5, Fig. 1d (4)). Specifically, participants aimed for the target, but once they initiated the movement (existed the target), the visual feedback of both the cursor and the target was turned off (vision OFF trials). Prior to each trial, participants were asked ‘How confident are you that you will hit the target?’ and responded (unlimited time) using a Likert scale (ranging from 1 to 5, with 5 indicating full confidence) in the centre of the workspace (Fig. 1e (1)). The Go cue was displayed 500ms after the confidence response and participants then performed the trial (with no visual feedback of the cursor or target (Fig. 1e (2)). The trial ended 2000ms after the go cue, when participants were asked ‘How confident are you that you hit the target?’ (CR2, Fig. 1e (3)) using the same Likert scale (ranging from 1 to 5) in the centre of the workspace (unlimited time). Participants completed 10 blocks of 5 trials (50 trials in total). Each block contained all targets (i.e., T100, T65, T35, T15, T5) which were presented in a random order. Therefore, participants completed 10 trials in each condition with each arm.

### Outcome variables

The 2D (x, y) position of the index finger was recorded at 1000 Hz by the KINARM Exoskeleton and was analysed ‘offline’ using Matlab (version R2019b, The MathWorks, Natick, MA, USA).

**Confidence Rating (CR1 and CR2)**. Confidence ratings ranged from 1 to 5, with 5 indicating full confidence, in both CR1 and CR2. Medians were calculated because one-sample Kolmogorov-Smirnov tests (Matlab function kstest) indicated that the data were not normally distributed.

**Success Rate (SR)**. Trial-based task success was operationalised as participants successfully hitting the target. Using the acquired 2D data (x, y) and the MATLAB function inpolygon we assessed if the participant hit the target at some point during the trial. SR represented the percentage of successful target hits.

### Analysis plan

*1)* Is confidence in achieving task success significantly different between the less and more affected upper limbs?

We performed a two-way repeated-measures ANOVA with ‘Arm’ (more and less affected limb) and ‘Task Difficulty’ (T100, T65, T35, T15, T5) as within factors and CR1 as the dependent variable. Post-hoc analysis included independent Wilcoxon Rank Sum Tests which were corrected for multiple comparisons using Bonferroni corrections while Cohen’s d was used to estimate effects sizes.

Additionally, we performed the same two-way repeated-measures ANOVA analysis as above using SR as the dependent variable, to assess differences in task success across limbs.

*2)* How is confidence about upper limb task success related to actual task performance?We performed a correlation between CR1 (median across all trials) and SR (% of successful target hits across all trials) independently for each limb.

*3)* Is confidence about task success and actual performance related to clinical measures including upper limb impairment?

To assess if confidence about task success or actual task success are a function of performance in clinical assessments, we ran independent correlations between both SR and CR1 and FMA scores. Furthermore, we ran the same analysis for FMA Sensory scores, HADS Anxiety sub scores, HADS Depression sub scores and NFI scores. Additionally, we performed a partial correlation between CR1 scores (prior reaching movement) to CR2 scores (post reaching movement) accounting for ‘Arm’ to assess if participants used post reaching feedback to update their confidence ratings.

## Results

### Study population

20 chronic stroke survivors (≥ 6 months from stroke onset) were recruited. The clinical and demographic characteristics are summarised in Table 1.

**Table 1:** Clinical and demographic characteristics participants

Characteristic	
Gender – male: female	13:7
Handedness (prior to stroke) – right: left	18:2
Affected upper limb – dominant: non-dominant	10:10
Age (years), mean (SD)	52.2 (11.9)
Time since stroke (months), mean (SD)	34.7 (37.2)
Fugl Meyer Assessment (modified FMA-UL - total 54 points; Ward et al., 2019), mean (SD)	28.2 (10.5)
Fugl Meyer Assessment Sensory (total 12 points) mean (SD)	10.8 (1.4)
Stroke Impact Scale (SIS-Hand – total score 100) mean (SD)	37.2 (23.2)
Hospital Anxiety and Depression Scale (HADS Anxiety - total score 21) mean (SD)	6.0 (2.96)
Hospital Anxiety and Depression Scale (HADS Depression - total score 21) mean (SD)	5.9 (3.1)
Neurological Fatigue Index (NFI - total score 34) mean (SD)	22.7 (11.7)

#### Confidence in achieving task success is significantly lower in the more compared to less affected upper limb

**Fig. 2:**
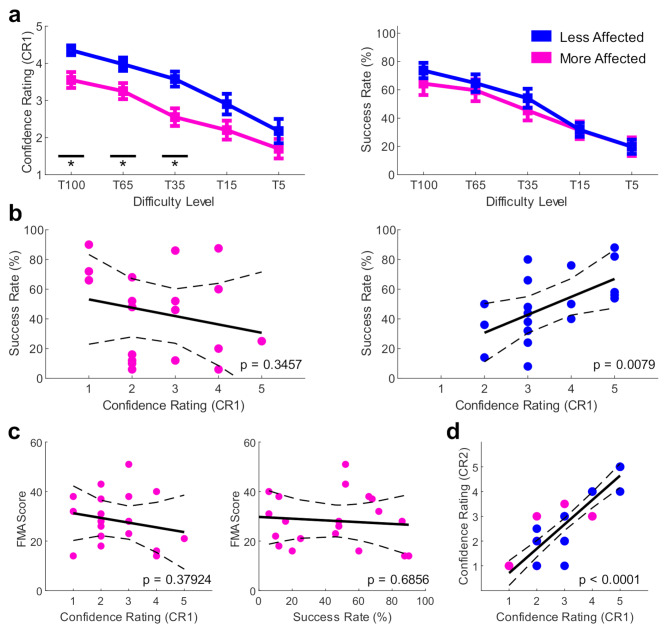
No evidence that actual and estimated upper limb motor ability correlate in the more affected arm only **a)** CR1 ratings were significantly lower in the more affected limb despite similar SR levels. *Left panel*: CR1 ratings and *Right panel*: SR for each difficulty level for both limbs. Averages and standard errors of the mean (SEM) were plotted as a line plot of the median values for each participant at each difficulty level (T100-T5) for both the more (pink) and less (blue) affected limb. * indicates significant differences between limbs. **b)** Correlation between confidence about task success (median CR1 across all trial) and success rate (% of successful trials across all trials, SR). *Left panel*: CR1 and SR were not correlated in the more affected limb. *Right panel*: But were significantly related in the less affected limb. **c-d)** A correlation analysis between *Left panel*: CR1 and FMA scores and *Right panel*: SR and FMA scores of the more affected revealed that neither are a function of motor ability in clinical assessments. **d)** Correlating CR1 and CR2 revealed a non-significant result, highlighting that participants did not change their rating after reaching for the target (i.e., did not use end position to update their confidence about task success).

Participants were less confident (CR1 - prior to the trial) about task success of their more affected upper limb (estimated upper limb motor ability) when compared to their less affected limb. Specifically, results from the two-way repeated-measures ANOVA revealed a significant main effect for ‘Arm’ (F = 22.61, *p* = 0.0001, η^2^ = 0.16, Fig. 2a, left figure) and ‘Task Difficulty’ (F = 146.40, *p* < 0.0001, η^2^ = 0.89). Importantly, we also found a significant interaction between ‘Arm x Task Difficulty’ (F = 18.62, *p* = 0.0003, η^2^ = 0.12). Post-hoc pairwise comparisons revealed that CR1 was significantly lower for the more affected limb for T100 (Z = -2.29, *p* = 0.0111, d = -1.01), T65 (Z = -2.30, *p* = 0.0356, d = -0.82), and T35 (Z = -2.85, *p* = 0.0111, d = -1.04) but not for the two most difficult levels T15 (Z = -1.82, *p* = 0.0864, d = -0.59) and T5 (Z = -1.07, *p* = 0.2843, d = -0.36). When looking at the differences in confidence between the more and less affected limb (i.e., Δ CR1) we found that 14/20 participants rated their confidence lower for the more affected limb (= 70%), with the remaining 6 participants not showing a difference between limbs.

Importantly, participants’ more and less affected limbs were equally successful at the reaching task, suggesting that SR was similar across limbs. Specifically, the two-way repeated-measures ANOVA did not reveal a significant main effect for ‘Arm’ (F = 0.56, *p* = 0.4629, η^2^ = 0.0.1, Fig. 2a, right figure) but did show a significant effect for ‘Task Difficulty’ (F = 63.29, *p* < 0.0001, η^2^ = 0.73). Importantly, we also did not find a significant interaction between ‘Arm x Task Difficulty’ (F = 0.28, *p* = 0.60, η2 < 0.01).

#### No evidence that actual and estimated upper limb motor ability correlate in the more affected arm only

Our results demonstrate that the relationship between actual and estimated upper limb motor ability has broken down only in the more affected limb. In fact, we found that CR1 and SR were not correlated in the more affected limb (ρ = -0.22, *p* = 0.3457, Fig. 2b, left panel), but were significantly related in the less affected limb (ρ = 0.58, *p* = 0.0079, Fig. 2b, right panel).

#### No evidence that actual and estimated upper limb motor ability correlate with clinical scores including upper limb impairment levels

To understand if upper limb estimated motor ability was related to impairment, we correlated CR1 and FMA scores and did not find a significant correlation (ρ = -0.21, *p* = 0.3792, Fig. 2c, left panel). Similarly, no significant correlation between SR and FMA scores were found (ρ = -0.10, *p* = 0.6856, Fig. 2c, right panel), highlighting that actual upper limb motor ability in this task is not a function of impairment. In line with these results, we did not find significant correlations for FMA Sensory (CR1: ρ = -0.15, *p* = 0.5306; SR: ρ = -0.12, *p* = 0.6334), HADS Anxiety (CR1: ρ = -0.46, *p* = 0.0612; SR: ρ = 0.04, *p* = 0.8910), HADS Depression (CR1: ρ = -0.21, *p* = 0.4143; SR: ρ = -0.07, *p* = 0.7899), and NFI scores (CR1: ρ = -0.27, *p* = 0.2568; SR: ρ = 0.07, *p* = 0.7840).

During the task no visual feedback of the cursor and target was provided to reduce availability of performance-based feedback, which could be used to update confidence about success rate. Yet, participants could still use their end position post-reach to infer if they were successful during a trial. A second confidence rating was included (CR2 – post target reach) to test if this feedback led to a change in confidence post-reach. We found a significant correlation between CR1 and CR2 (ρ = 0.87, *p* < 0.0001, Fig. 2d), highlighting that participants did not change their rating after reaching for the target.

## Discussion

We found that chronic stroke survivors’ confidence about task success was lower in their more affected compared to their less affected limb despite similar task success when using either limb. Furthermore, actual and estimated upper limb motor ability did not correlate in the more affected limb, whilst they did in the less affected limb. This suggests that in the present sample, stroke survivors did not adjust their perception of upper limb ability to match actual upper limb ability post-stroke, which may be a key factor underlying daily life limb use in chronic stroke [6–9, 12].

Importantly, our results highlight that actual and estimated upper limb motor abilities can dissociate in chronic stroke independently from upper limb impairment level. Lacking confidence in affected upper limb abilities should, therefore, be considered as a therapeutic target. Specifically, targeted neurorehabilitation strategies that centre around confidence coaching could be implemented to increase the incorporation of the more affected upper limb into activities of daily living.

Past research on a possible mismatch has produced inconsistent results [12–21]. Within this context, it is important to highlight three factors that may have contributed to the diverging results.

1) While some used standardised questionnaires such as the SIS-Hand or ABILHAND to assess confidence about UL motor abilities, others used visual analog scales for ratings on how confident a patient is in achieving a specific task goal [12–20]. Similarly, some studies used FMA or ARAT scores to determine actual motor ability, while others used virtual reaching tasks or assessments of grip strength [12–20]. The heterogeneity of assessment tools used may account for the diverging results found in the literature. Importantly, only one study employed a task design in which confidence about task performance was directly compared to task success on the same task [21].

2) Most studies did not account for the fact that confidence, which is believed to be a personality trait [25, 26], may differ across stroke survivors. Specifically, some survivors may have higher confidence irrespective of the task or question which may affect the correlation between perceived and observed motor abilities.

3) Providing performance-based feedback has the potential to shape confidence about UL motor ability because it provides a difference signal of how much estimated and actual diverge [27]. Consequently, estimation of UL motor abilities can be re-evaluated after provision performance-based feedback which may obscure the actual extend of the mismatch.

In the present study actual and estimated motor ability was assessed on the same experimental task using a within-participant design without provision of performance-based feedback in a stroke survivor sample with a wide range of impairment. Using such a design has several advantages: it allows for a direct comparison between actual and estimated motor abilities (increased consistency), assesses task-specific rather than generalised confidence (increased specificity), and it accounts for confidence changes that occur because of changes in task difficulty (increased sensitivity). It revealed a clear mismatch between actual and estimated motor abilities in chronic stroke and may suggest that different task designs may occlude such a finding.

Previous approaches defined non-use as minimal impairment (FMA-UE scores > 50/66) coupled with low self-reported day to day upper limb use (Stroke Impact Scale (SIS-Hand) < 75/100) [12–14]. We found that 3 of our participants (16.7%, no SIS-Hand data for 2 participants) had non-use by this definition. However, our design allowed us to study patients with higher levels of upper limb motor impairment and overall revealed that 14 (70%) participants had a mismatch between actual and estimated upper limb motor ability. A mismatch in our design is based on the differences in confidence between both limbs considering that both achieve similarly successful at the task. Our results highlight that a lack of confidence in upper limb ability is not reserved for those with minimal impairment but can be seen in those with moderate to severe impairment too. This suggests that improving confidence in affected upper limb ability could be a therapeutic target for a wide range of stroke survivors.

A lack of confidence refers to an individual’s belief in not meeting specific situational demands and is often credited as the reason behind a drop in performance in people with equivalent skills or abilities [28]. Such a situational lack of confidence is, therefore, state-specific and can be contrasted from trait-specific confidence, which is believed to be a personality trait (Stankov and Lee, 2008; Burns et al., 2016). Here, we account for inter-subject differences in trait confidence by using a within participant design. Yet, state confidence can be affected by other factors such as anxiety, depression and/or fatigue [29]. We did not find a correlation between confidence ratings of the more affected limb with ratings on the sub scores of the Hospital Anxiety and Depression Scale (HADS) and the Neurological Fatigue Index (NFI). Note here, that the HADS Anxiety scores were close to significance, which may suggest that anxiety modulates state confidence. Furthermore, confidence ratings were not modulated by the presence of sensory loss.

In the present, highly controlled experiment we minimised sensory load and interference, deweighted the participants’ arm using the KINARM Exoskeleton, and focused on a reaching task that did not require hand movement. This enabled us to determine the differences between estimated and actual motor abilities in chronic stroke. However, moving forward, future research needs to translate the present experiment into more complex, real-world contexts in which sensory feedback, environmental demands, and task consequences may vary. This will provide a more comprehensive understanding of how confidence about UL motor abilities affects chronic stroke survivors in daily life.

Accurate estimation of one’s capabilities is crucial for successfully engaging in everyday life activities. This is particularly important in the context of chronic stroke, where a lack of confidence, or underestimation of abilities, has been shown to predict non-use of affected limbs. Targeting the mismatch between estimated and actual upper limb motor abilities could significantly improve rehabilitation outcomes. Future research should investigate this mismatch through the lens of psychological models and theories to uncover the underlying mechanisms contributing to the observed lack of confidence. This could promote the development of targeted interventions to increase confidence and the use of affected limb.

In summary, we found clear evidence for a mismatch between actual and estimated upper limb motor ability for the more affected upper limb in a wide range of chronic stoke survivors. Our findings suggest that a lack of confidence in affected upper limb motor ability may be prevalent across impairment levels. We suggest that testing for a mismatch between actual and estimated in chronic stroke may highlight those at risk of developing non-use of the affected upper limb and point towards potential therapeutic opportunities.

## Data Availability

The data that support the findings of this study are available on request from the corresponding author. The data are not publicly available due to them containing information that could compromise the privacy of research participants.
